# Development of an Injectable Shear-Thinning Nanocomposite Hydrogel for Cardiac Tissue Engineering

**DOI:** 10.3390/gels8020121

**Published:** 2022-02-14

**Authors:** Samaneh Soltani, Rahmatollah Emadi, Shaghayegh Haghjooy Javanmard, Mahshid Kharaziha, Abbas Rahmati, Vijay Kumar Thakur, Saeid Lotfian

**Affiliations:** 1 Biomaterials Research Group, Department of Materials Engineering, Isfahan University of Technology, Isfahan 84156-83111, Iran; samaneh.soltani34@yahoo.com (S.S.); remadi@iut.ac.ir (R.E.); kharaziha@cc.iut.ac.ir (M.K.); 2 Applied Physiology Research Center, Cardiovascular Research Institute, Department of Physiology, School of Medicine, Isfahan University of Medical Sciences, Isfahan 81746-73461, Iran; shaghayegh.haghjoo@gmail.com; 3 Department of Chemistry, University of Isfahan, Isfahan 81746-73441, Iran; a.rahmati@sci.ui.ac.ir; 4 Biorefining and Advanced Materials Research Center, Scotland’s Rural College (SRUC), Kings Buildings, Edinburgh EH9 3JG, UK; 5 School of Engineering, University of Petroleum & Energy Studies (UPES), Dehradun 248007, India; 6 Faculty of Engineering, University of Strathclyde, Glasgow G4 0LZ, UK

**Keywords:** shear-thinning, dual-cross linked hydrogels, mesenchymal stem cells, cardiac tissue engineering

## Abstract

Bone marrow-derived mesenchymal stem cells (MSCs) offer a promising therapeutic method for cardiac tissue regeneration. However, to monitor the fate of MSCs for tissue repair, a better stem cell delivery carrier is needed. Developing a unique injectable and shear-thinning dual cross-linked hybrid hydrogel for MSC delivery for cardiac tissue engineering is highly desirable. This hydrogel was synthesised using guest: host reaction based on alginate-cyclodextrin (Alg-CD) and adamantane-graphene oxide (Ad-GO). Here, the role of macromere concentration (10 and 12%) on the MSC function is discussed. Our hybrid hydrogels reveal a suitable oxygen pathway required for cell survival. However, this value is strongly dependent on the macromere concentrations, while the hydrogels with 12% macromere concentration (2DC12) significantly enhanced the oxygen permeability value (1.16-fold). Moreover, after two weeks of culture, rat MSCs (rMSCs) encapsulated in Alg-GO hydrogels expressed troponin T (TNT) and GATA4 markers. Noticeably, the 2DC12 hydrogels enhance rMSCs differentiation markers (1.30-times for TNT and 1.21-times for GATA4). Overall, our findings indicate that tuning the hydrogel compositions regulates the fate of encapsulated rMSCs within hydrogels. These outcomes may promote the advancement of new multifunctional platforms that consider the spatial and transient guidelines of undifferentiated cell destiny and capacity even after transplantation for heart tissue regeneration.

## 1. Introduction

Ischemic coronary disease is a global medical problem that influences a large number of individuals. Every 34 s, one person dies in the United States due to cardiovascular disease [1]. Current heart failure treatments, including therapeutic and surgical procedures, could enhance quality and length of life. Even though heart attack deaths have decreased, successful therapy for ventricular dysfunctions is still lacking [2]. Nowadays, cellular treatments for ischemic myocardium have risen as a promising approach. Skeletal myoblasts, endothelial begetter cells, embryonic stem cell myocytes, embryonic myocytes, and mesenchymal stem cells (MSCs) have been used in experimental designs [3,4,5,6]. Since MSCs can differentiate into cardiomyocytes, they are valuable sources for cardiac cell therapy. Previously, MSCs have been evaluated clinically (for cardiac regeneration) and do not create any serious problems [4,5]. One of the most challenging aspects of using MSCs was optimising cell injection, dispersion, and fattening of cells for therapeutic purposes. Furthermore, only around 1% of the cells are retained after direct cell injection into the myocardium [7,8,9,10]. Some critical factors that lead to poor cell maintenance were included irritation, mechanical removal of beating cardiovascular cells and coronary arteries, and leakage of cell suspensions from the injection locations [11,12,13,14,15]. Most cell death happens just after a few days. As a result, a delivery and stabilisation system should support cell retention and transplant over long periods of time [16].

Recently, the encapsulation of MSCs in hydrogel-based biomaterials has been widely described for cardiac tissue regeneration and repair [16,17,18,19]. Hydrogels are frequently utilised to simulate the physical and chemical properties of the extracellular environment of stem cells and influence phenotypes and cell proliferation [20,21,22]. In this regard, chemical properties, electrical conductance, mechanical stiffness, and elasticity of hydrogels noticeably affect the destiny and fate of the encapsulated stem cells [23]. Alginate (Alg) has been widely applied for cardiac cell encapsulation [24,25]. Etter et al. [26] developed methacrylate Alg microspheres for MSC encapsulation. However, methacrylate alginate hydrogel could not provide appropriate mechanical and rheological properties for injecting MSCs. Various studies have developed hybrid hydrogels based on Alg to overcome this challenge. For example, Choe et al. [27] developed reduced graphene oxide (rGO)/Alg microgels used to encapsulate MSCs and recover infarcted hearts. In vivo tests with serious MI models showed that the administration of MSCs using rGO/Alg microgels increased therapeutic effectiveness, substantially reducing the infarcted area and improving heart function. However, high shear stress and cell leakage during hydrogel injection have relied on transported stem cells’ low viability and activity. Recently, we developed a shear-thinning injectable hydrogel based on Alg and GO [28]. The dual-crosslinked Alg-GO hydrogel was developed by guest-host and ionic interactions (Ca^2+^) between adamantane-modified GO (guest macromere; Ad-GO) and β-cyclodextrin-modified Alg (host macromere; Alg-CD). We found that the dual-crosslinked hydrogel with 12% macromeres concentration and 1:2 host: guest macromere ratio (2DC12) revealed high strength (4.0 ± 0.7 MPa) and toughness (11.2 ± 0.1 MJ/m^3^). Notably, less than 20% weight loss was indicated for 2DC12 after 20 days of incubation in saline. We also found that the dual-crosslinked Alg-GO hydrogel could support fibroblast cell proliferation seeded on the hydrogel. On the other hand, it is expected that the shear-thinning of the dual-crosslinked Alg-GO hydrogel may decrease mechanical damages to cells during infusion. However, the ability of the dual-crosslinked Alg-GO hydrogel for cell encapsulation has never been investigated. In addition, the ability of this hydrogel to control MSC differentiation into cardiac cells has not yet been considered.

This research aimed to study the role of dual-crosslinked Alg-GO hydrogel to modulate the differentiation of MSCs. Moreover, the role of various concentrations of Alg: GO on the cell functions is studied. In this study, we chose rat-MSCs (rMSCs) since they are easily accessible and show high vascularity [29]. We assumed that combining Alg-GO hydrogel and rMSCs will stimulate rMSCs to differentiate into myocytes used in myocardial ischemia treatment.

## 2. Results and Discussion

### 2.1. Characterisation of Alg-GO Hydrogels

**Figure 1:**
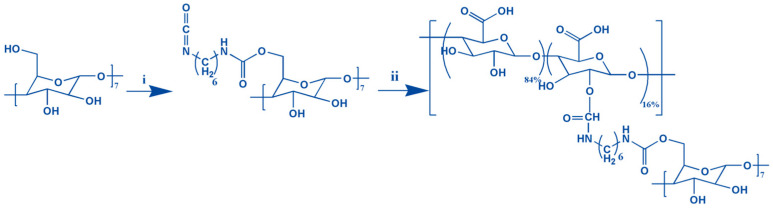
The formation of the Alg-CD schematic shows synthesis from the combination of CD-hexamethylene diisocyanate (i) and alginate (ii).

Graphene oxide-adamantane and alginate-β-cyclodextrin are the macromers for the fabrication of alginate/graphene oxide-based smart shear-thinning nanohybrid hydrogels. The controlled esterification reaction between the modified adamantane and the graphene oxide alcohol groups resulted in forming a thin layer of adamantane on the surface of the graphene oxide nanosheets. Furthermore, the two-step process of making alginate-β-cyclodextrin by hexamethylenediamine isocyanate is done (Figure 1).

**Figure 2:**
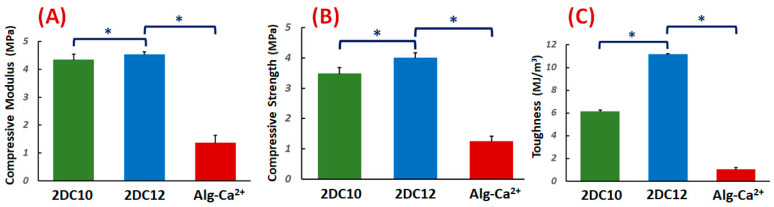
Mechanical properties of nanohybrid Alg-CD + Ad-GO hydrogels: (**A**) compressive modulus (at 80% strain), (**B**) compressive strength and (**C**) toughness of DC hydrogels (*: *p* < 0.05).

The compressive characteristics of the DC hydrogels were investigated. Figure 2A–C demonstrate the compressive modulus, strength, and toughness. The compressive modulus of DC hydrogels was altered with changing macromeres concentration (*p* < 0.05) (Figure 2A). These data illustrated that at high macromere concentration (2DC12), the strong interaction between Ad-GO and Alg-CD hydrogels resulted in enhanced compressive modulus. It could be due to the influential role of GO nanosheets in effectively transferring the stress to physical cross-linking bridges.

Moreover, the compressive strength of hydrogels was considerably promoted for 2DC12 samples with increasing macromere concentration (2DC12 sample) (*p* < 0.05), demonstrating the influential role of the cross-linking process to increase the mechanical strength. The significant mechanical properties of GO nanosheets, the robust interaction between the Ad groups of Ad-GO with Alg-CD, and the catechol groups of Alg backbone with Ca^2+^ ions endorsed the mechanical strength nanohybrid hydrogels. The mechanical strength was enhanced by enhancing macromere concentration. Finally, we observed that the concentration of macromeres significantly enhanced the hydrogel toughness (*p* < 0.05). It could be demonstrated that enhanced physical cross-linking of hydrogels led to considerably improved mechanical toughness [28,30].

**Figure 3:**
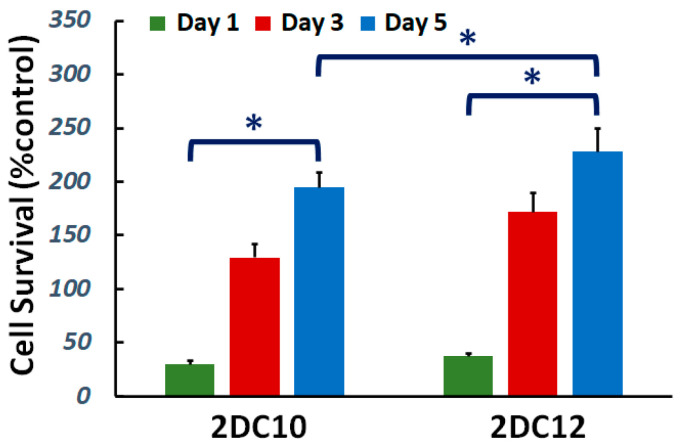
Cytocompatibility of nanohybrid alginate-GO hydrogels: effect of concentration of macromers on the cell viability (*: *p* < 0.05).

Cytocompatibility of nanohybrid hydrogels is studied. The viability of L929 cells cultured on the samples was investigated. Figure 3 demonstrate cell viability enhanced with increasing culture time for all samples. Furthermore, after five days of culture, cell survival increased. Furthermore, with raising macromere concentration, the cell survival noticeably improved. Alg-GO hydrogels were developed consuming a dual physical cross-linking process; host-guest (GH) reaction and ionic cross-linking. Raman spectroscopy was applied to characterise the GOPD nanoparticle to distinguish ordered and disordered crystal structures of carbon, and the result was presented in the previous publication [31]. The G band peak at about 1597 cm^−1^ corresponded to the vibration of the *sp*^2^-bonded carbon atoms in a two-dimensional hexagonal lattice, while a D band peak at about 1325 cm^−1^ specified disorder the Raman of the GO. It has been indicated previously that the disorder in the GO nanosheets was originated from defects associated with vacancies, grain boundaries and amorphous carbon species [32,33]. Our findings showed that the ID/IG of GO was about 0.95 [31].

**Figure 4:**
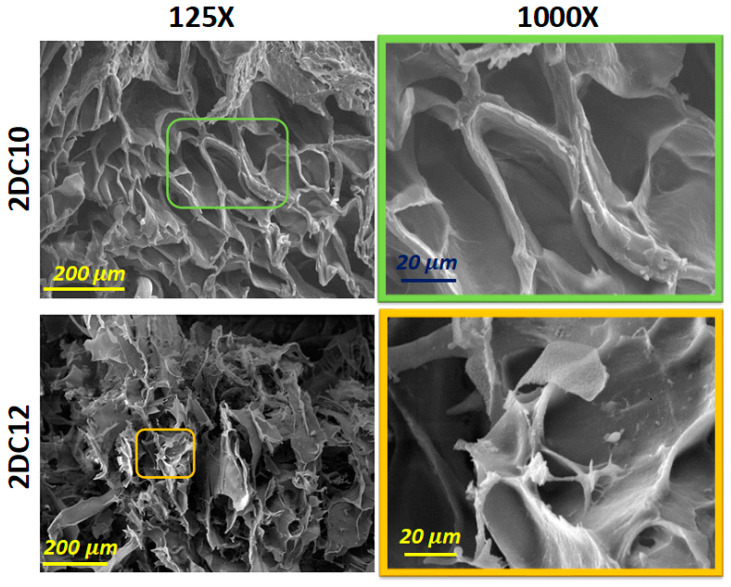
SEM micrograph of 2DC10 and 2DC12 hydrogels.

Scanning electron microscopy (SEM) images of these hybrid hydrogels developed using two composition concentrations of Ad-GO and Alg-CD macromeres (2DC10 and 2DC12) are presented in Figure 4. Our results show that all nano-hybrid hydrogels, depending on the concentration of the macromere, consist of three-dimensional structures with different pore sizes and interconnected pore distributions. All of the samples displayed interconnected pore networks. This interconnected porous network might help cells survive and proliferate by facilitating nutrition and waste product movement. According to Figure 4, 2DC12 indicated the greater pore size and more interconnected pores, affecting its oxygen permeability (OP) value and, as a result, cell function and viability, especially for cardio differentiation.

**Figure 5:**
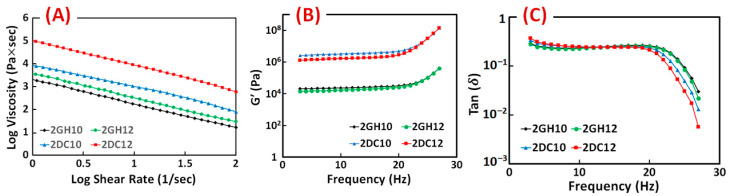
(**A**) Viscosity, (**B**) storage modulus (G′) and (**C**) loss tangent (tan (δ)) of GH and DC hydrogels composed of different macromere concentrations and guest macromere ratios.

The rheological characteristics of the GH and DC hydrogels were investigated. The results show the entanglement of host-guest macromeres changed the viscosity of GH hydrogels, which was due to the hydrophobic interactions between Ad and CD groups. Correspondingly, shear-thinning behaviour is significantly enhanced by incorporating Ca^2+^ ions in (Alg-CD + Ad-GO) GH hydrogel. For all the shear rates, the results of DC hydrogels exhibited higher viscosity than GH hydrogels [28]. As the shear rate increased, the viscosity of samples reduced, which supported the sample’s viscoelastic behaviour (Figure 5A). The relation between the storage modulus across the frequency is shown improvement with increasing the macromere concentration or ratio through the frequency. The behaviour of the loss moduli is similar. Furthermore, the loss tangent (tan (δ) = G″/G′) decreased as the frequency increased, confirming the formation of rigid structures. It can happen when cross-linking density changes, altering the rheological properties. In particular, a dramatic decrease in the loss tangent was detected in the DC hydrogels. The significant interactions among hydroxyl groups of Alg and Ca^2+^ ions accrued in hydrogels, resulting in enhanced elastomeric behaviour. DC hydrogels selectively involved Ca^2+^ ions in this process and demonstrated shear-thinning and rapid self-healing behaviour [28].

**Figure 6:**
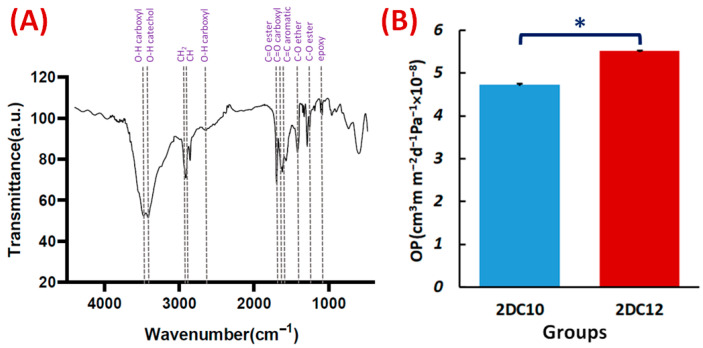
Characterisation of Alg-GO hydrogel: (**A**) FTIR spectrum of Alg-GO (2DC12) hydrogel. (**B**) The OP properties of 2DC10, and 2DC12 hydrogels (*n* = 3) (* *p* < 0.05).

The FTIR spectra of the Alg-GO hydrogel (Figure 6A) also exhibited Ad-GO and Alg-CD macromeres peaks. In that order, ester groups of Ad and GO at 1710 cm^−1^ and 1285 cm^−1^ were identified with the C=O and C-O bonds. The peaks at 3420, 1540, 1285, and 1140 cm^−1^ were also identified as GO distinctive peaks [30,34]. Three peaks on the CD were also found at 3405, 2920, and 1250 cm^−1^, respectively, corresponding to the catechol, C-H, and C-O stretching of ether. The FTIR spectra of the Alg-GO hydrogel (Figure 6A) also exhibited Ad-GO and Alg-CD macromeres peaks that validated the efficient GH reactions between hydrophobic groups of CD and Ad in macromeres. As a result, the primary hydroxyl group of Alg plays a main character in the ionic cross-linking with Ca^2+^ ions [28].

Hydrogel materials are appealing scaffolds for cell encapsulation because they have good biocompatibility and excellent permeability for oxygen, nutrients, and other water-soluble metabolites. One of the essential properties of hydrogels is their OP value. Interestingly, OP value is affected by interconnected pores and pore size. This may help the cells contained within the hydrogel to survive and grow [35]. According to Figure 6A, 2DC12 hydrogel with an OP value of 5.51 × 10^−8^ ± 0.01 cm^3^ m m^−2^ d^−1^ Pa^−1^ shows the excellent oxygen route. As more macromeres were added, the porosity of hydrogels increased, influencing the OP values of the hydrogels and improving the OP values (1.6-folds). Furthermore, higher oxygen solubility in hydrophilic hydrogels is thought to promote oxygen transport inside the hydrogels [36].

### 2.2. Immunocytochemical Analysis

#### Expression of Cardiac Markers

**Figure 7:**
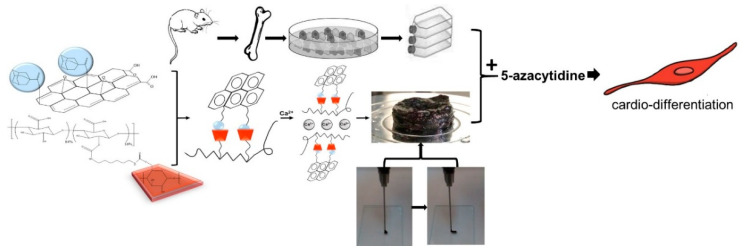
The schematic shows the differentiation of rMSCs to cardiac myocytes on injectable shear-thinning Alg-GO hydrogel (with GH and DC interactions) scaffolds.

According to Figure 7, nanohybrid hydrogels were developed via GH interactions between Ad-GO and Alg-CD macromeres. Then, we incorporated Ca^2+^ within Alg-GO hydrogels to develop ionic cross-linked hydrogels. As a result, an injectable shear-thinning hydrogel with desirable rheological and mechanical properties for soft tissue engineering was generated [28]. The capacity of Alg-GO hydrogels for rMSCs encapsulation was then investigated. It was expected that rMSCs differentiate to myocytes via 5-azacytidine as a growth factor.

Following myocardial damage, GATA4 is required for cardiac myocyte viability and hypertrophic response [37,38]. It has been shown that GATA4 directly promotes BCL2 expression in cardiac muscle cells in vitro [39]. Troponins, which are muscle-specific proteins, also make up the contractile filaments and are essential in contraction regulation [40,41]. According to Figure 7, TNT and GATA4 were expressed by rMSCs encapsulated in Alg-GO hydrogels. On the contrary, as a control group, rMSCs encapsulated in Alg hydrogels were unsuccessful in exhibiting any positive staining.

**Figure 8:**
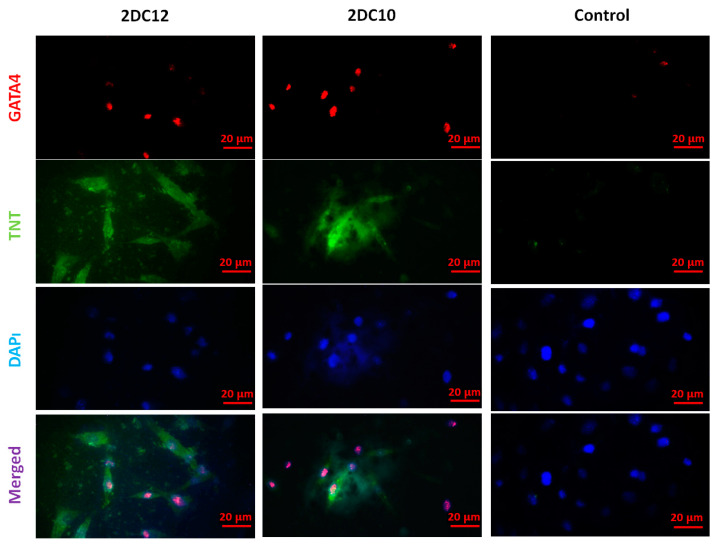
Immunofluorescent microscopy images of the cardiac assay of the rMSC after two weeks of culture on various hydrogels. The rMSCs express the contractile protein, cardiac troponin T (TNT) (green), as well as the cardiac-specific transcription factor, GATA4 (red). Cell nuclei were stained by DAPI (blue). The scale bar is 20 μm.

Furthermore, encapsulated rMSCs in 2DC12 hydrogel with more macromeres revealed higher levels of TNT and GATA4 expression. The microscopic images were analysed, and the results are presented in Figure 8. Results indicated that the encapsulated rMSCs in 2DC12 hydrogel with more macromeres had higher TNT and GATA4 expression levels, which seems to be due to the desired mechanical properties of Alg-GO hydrogels control cell functions [28]. It might be linked to the beneficial effects of GO nano-sheets. Previous studies also showed that GO nano-sheets might modify substrates’ surface properties and stiffness, promoting cell activity [42,43]. Chen et al. [44] discovered that graphene and its derivatives like GO Fibroblasts could cultivate cell adhesion and proliferation. In the incomparable investigation, Kang et al. [45] identified considerable development in the cell tasks after the combination of GO to the polymeric construction owing to helped stiffness of the soft environment. Moreover, Zhang et al. [46] detected that a combination of adjusted GO into polymeric hydrogels enhanced its mechanical features and stimulated cell viability. About earlier results, a cell’s fate could be organised by many chemical and physical characteristics of the surroundings, called cell niche [42,47]. Among them, mechanical signals and especially stiffness played a critical part to control cell tasks [48]. The results demonstrate that mouse mesenchymal stem cells encapsulated in 2DC12 hydrogel with higher macromers had higher levels of TNT and 4GATA expression, which could be because of the desired mechanical properties of alginate/graphene oxide hydrogels, which control cell functions [37]. It is related to the beneficial effects of nano-graphene oxide sheets. According to the previous studies, nano-graphene oxide sheets may alter substrates’ surface properties and stiffness and increase cellular activity [41]. Our results illustrated that the inclusion of GO in the composition of Alg-GO hydrogels increased mechanical properties and improved cell adhesion and diffusion. Additionally, hydroxyl groups on the ground surface can successfully raise adhesion sites to interest proteins in the culture medium by electrostatic interaction, thereby improving cell adhesion and diffusion.

**Figure 9:**
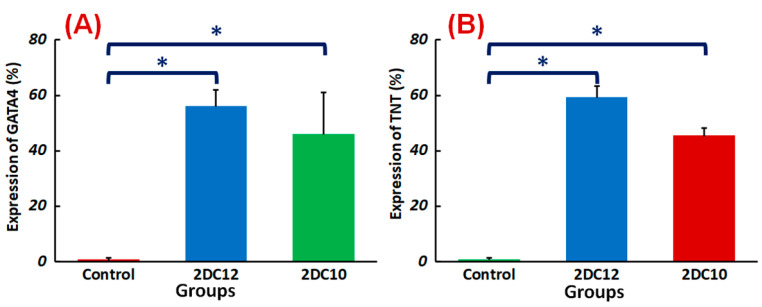
Differentiation of rMSCs encapsulated Alg-GO hydrogels: quantified expressions of (**A**) GATA4 (%) and (**B**) TNT (%), measured after two weeks of culture (*n* = 3) (* *p* < 0.05).

Despite advancements in myocardial infarction (MI) treatment, recent pharmacological and interventional therapies are having difficulty regenerating the damaged myocardium [49,50]. Due to MSC’s strong proliferation, flexibility, and homing potential, MSC transplantation to the ischemic region might be a viable therapeutic for enhancing the left ventricular role after a heart attack [51]. This study shows that the Alg-GO hydrogel is a promising choice for cardiac tissue engineering. TNT and GATA4 were represented by rMSCs encapsulated in Alg-GO hydrogels. TNT and GATA4 expression were also higher in encapsulated rMSCs in hydrogel formulations with higher macromere concentrations (Figure 9). The importance of the medium in determining the rest and fate of encapsulated stem cells was supported by these findings.

To value the controlling result of including scaffolds in the predictions of MSC’s fate, inclusive MSCs in the general media were cultured containing 5-azacytidine. We found that presenting the MSCs for cardio-differentiation with an attractive scaffold is essential for regenerating high-quality tissues. Generally, our results suggest that the physicochemical features of hydrogel biomaterials and the presence of attractive signals may affect the associated performance of rMSCs. These parameters will determine the fate of encapsulated MSCs against the target phenotype.

## 3. Conclusions

This study outlines a cardio regeneration technique focused on in vitro experiments that demonstrate rMSC’s ability to react to matrix physic-chemical properties (like interconnected pores, mechanical and rheological properties) and inductive signals like growth factors (5-azacytidine). Our study established an injectable shear-thinning hydrogel based on functionalised alginate (Alg) and graphene oxide (GO) using ionic and guest: host interactions. These hydrogels were created via a dual-crosslinking method, transforming them into one-of-a-kind biomaterials with exceptional capabilities. By boosting Alg-GO shear-thinning action, the inclusion of functionalised macromeres with Ad: CD complex allowed it to inject efficiently. These hydrogels revealed a porous network with interconnected pores that caused excellent OP. In short, our results propose that altering the hydrogel structure can be used to modify the fate of hydrogel-encapsulated rMSCs. In conclusion, immunocytochemical comparisons showed that rMSCs could differentiate into cardiomyocyte-like cells containing Alg-GO hydrogel scaffolds. These results may lead to the development of new multitask scaffolds that allow three-dimensional and progressive regulation of stem cell fate and function even after transplantation.

## 4. Materials and Methods

### 4.1. Isolation and Culture of rMSC

In this study, rMSCs were acquired from 60–80 g adult male Sprague-Dawley rats. Primary, MSCs were isolated according to Wakitani et al. [29] study from Sprague-Dawley rats, with a few changes. Briefly, male Sprague-Dawley rat’s femora and tibiae were extracted, and their soft tissues were carefully detached that the marrow preparation was free of myogenic precursors. Scissors were used to cut two ends of the bones (femora and tibiae). Using needles loaded with growth medium, bone marrow plugs were removed from bones, and bone marrow cells were centrifuged twice before being re-introduced into the growth medium. In this way, the cells were suspended again and put in tissue culture vials in a 6 mL growth media (α-MEM culture medium, supplemented with 10% (*v*/*v*) FBS, freshly prepared AA (50 μg/mL), 50 IU L^−1^ penicillin, 2 mM L-glutamine, 20 μg mL^−1^ streptomycin, 0.3 μg mL^−1^ fungizone, 50 μg mL^−1^ gentamycin sulphate, 10 mM β-GP, 10^−8^ M dexamethasone) for 5 days. After three days, the environment was changed, and the non-adherent cells were removed. Twice a week, changing of the culture medium was done. The culture flasks nearly merged, and the adherent cells were discharged with 0.25% trypsin from the dishes. It all happened around 7–10 d after seeding (Gibco Laboratories, Grand Island, NY, USA). They split 1:2 and seeded into fresh culture flasks. The cells were cultured in Dulbecco’s modified Eagle’s medium (DMEM; Gibco Laboratories, Grand Island, NY, USA) and 10% fetal calf serum (FCS; Hyclone, Logan, UT, USA) when every 3 d, the medium was changed once. Finally, the cells were developed with 5% CO_2_ at 37 °C in a humidified atmosphere. The third to the fifth passage cells were used in all subsequent studies.

### 4.2. Synthesis of Alg-GO Hydrogels

The Ad-GO and Alg-CD macromeres were synthesised according to our previous study [28]. Initially, Ad-GO was synthesised using a simple esterification process via the interaction between GO nanosheets (Nanosany Co, Mashhad, Iran) and Ad. Initially, 2 wt.% 1-adamantane carboxylic acids (Sigma-Aldrich, Burlington, MA, USA) was dissolved in propylene carbonate, and consequently, acetyl chloride (7.8 mL, Sigma-Aldrich) was mixed with it overnight. Consequently, GO nanosheets (2 g) were dispersed in the above solution for 12 h at 60 °C, and therefore, the products were quickly washed with double distilled water (DDW) and dried in a vacuum. Urethane bonding was also used to create Alg-CD. First, β-CD (1 mol, Sigma-Aldrich) was dissolved in dimethyl sulfoxide (DMSO, Sigma-Aldrich), and then hexamethylene diisocyanate (7 mol, Sigma-Aldrich) was blended among it. The Alg solution in minimal DMSO was mixed with the solution via syringe at 60 °C for 24 h. The solution was sieved against DDW lyophilised after dialysis for seven days.

Alg-GO nano-hybrid hydrogels were made using a mixture of Alg-CD and Ad-GO macromeres at a 1:2 macromere ratio and different macromere concentrations (10% and 12%). Each macromere (Ad-GO and Alg-CD) was primarily dissolved in DDW at room temperature at the necessary concentrations (10% and 12%) and then stirred for 2 min and centrifuge for 5 min to remove trapped air and use guest-host (GH) assembly process to make Alg-GO hydrogel. Finally, an ionic cross-linking process was performed using a 0.01 M CaCl_2_ solution to prepare a double cross-linked (DC) hydrogel.

### 4.3. Characterisation of Alg-GO Hydrogels

The compressive mechanical properties of nano-hybrid hydrogels are investigated using a tensile instrument (H25KS; Hounsfield Test Equipment Ltd., Salfords, UK) with a load cell capacity of 500 N and a strain rate of 1 mm/min. The samples are prepared in 10 mm diameter and 20 mm thickness (*n* = 3). L929 fibroblasts (Royan Institute, Tehran, Iran) were used to study the cytocompatibility of nanohybrid hydrogels. L929 cells were cultivated in Dulbecco’s Modified Eagle Medium (DMEM, Gibco, Waltham, MA, USA) augmented by 10% (*v*/*v*) fetal bovine serum (FBS, Gibco, Waltham, MA, USA) and 1% (*v*/*v*) antibiotic-antimycotic solution (penicillin, streptomycin, amphotericin, and gentamycin, Gibco, Waltham, MA, USA) under standard condition (37 °C, 5% CO_2_/95% humidity). The cells were separated by trypsin-EDTA solution (0.25%) after getting 80–90% confluency, and viable cells were counted by trypan blue assay. The disk-shaped nanohybrid hydrogels (*n* = 3) were prepared, washed with PBS, immersed in 70% (*v*/*v*) ethanol for 30 min, and exposed to UV light for two hours. The cells were seeded on the samples at a 20 × 10^3^ cells/well density and cultured at 37 °C, 5% CO_2_, and 95% humidity. At the specific time points (1, 3, and 5 days), the cell-seeded hydrogels were washed with PBS and consequently were incubated with MTT (3-(4,5-dimethylthiazol-2-yl)-2,5-diphenyltetrazolium bromide) solution in a fresh medium (5 mg/mL). DMSO was added to dissolve formazan crystals after four hours of incubation at 37 °C in 5% CO_2_. Then, the mixtures were pipetted into a 96-well plate, and the absorbance of the DMSO solutions was evaluated at 545 nm using a microplate reader (BioTek, Winooski, VT, USA).

The chemical properties of Ad-GO and Alg-CD were studied using the Fourier transform infrared spectroscopy (FTIR, Bruker, Billerica, MA, USA). The microstructure of the Alg-GO hydrogels was studied using scanning electron microscopy (SEM, XL30, Philips Electron Optics B.V., Eindhoven, The Netherlands). A PERME OX2/230 (Labthink, Jinan, China) was used to measure the hydrogels’ oxygen permeability for three replications at 25 °C and 0% RH. Briefly, the hydrogels were put in a 50 cm^2^ open testing area chamber. On one side of the film, nitrogen flowed at a pace of 10 mL/min, while oxygen flowed at a rate of 60 mL/min. The OP was determined using the following equation (Equation (1)) [52]:

(1) $$\mathrm{OP}=\frac{\mathrm{OTR}\times \mathrm{FT}}{\Delta p}$$

where OP is oxygen permeability, OTR is oxygen transmission rate (cm^3^/m^2^ d), FT is the film thickness (m), and Δp is the partial pressure of oxygen.

### 4.4. Development of rMSCs Encapsulated Alg-GO Hydrogel and Cardiac Differentiation

The cells are added during the formation of hydrogels. They are added to guest-host hydrogels, and after that, CaCl_2_ solution is mixed to make dual cross-link hydrogel. Worth noting that the guest-host hydrogel is injectable, but dual cross-linked hydrogel is not injectable. 1 × 10^6^ rMSCs were encapsulated in 1 mL of each hydrogel composition or Alg hydrogel. As a control group, pure Alg hydrogel was employed. To induce cardiac differentiation, nearly cell-encapsulated hydrogels were precipitated with 5 μm 5′-azacytidine (Sigma-Aldrich GmbH, Hamburg, Germany) in differentiation medium (Iskov’s Modified Dulbecco Medium/Hames F12 (1:1), IMDM; Invitrogen, Darmstadt, Germany) L-glutamine, non-essential amino acids, 2% FBS (Sigma-Aldrich GmbH, Hamburg, Germany), Supplemented with 10^−4^ M ascorbic acid (Sigma Aldrich, Germany), and 1% insulin-transferrin-selenium (Invitrogen GmbH, Karlsruhe, Germany) for three weeks [53]. The rheological properties of nanohybrid hydrogels were studied utilising a rheometer operated with a 20-mm diameter cone and plate geometry at a gap of 27 μm. A flow test was performed to determine the viscosity of GH and DC cross-linked hydrogels. In contrast, the shear rate was adjusted from0.01 s^−1^ to 100 s^−1^ at 25 °C. These tests were performed for both GH and DC hydrogels to test the effect of macromere concentrations and guest macromere ratios on the rheological properties [28].

### 4.5. Immunocytochemistry of rMSC

The cell encapsulated hydrogels were rinsed with PBS and fixed with 3.7 wt.% formaldehyde (Roth GmbH & Co. KG, Nuremberg, Germany) for 10 min at 25 °C for a confluent culture. Consequently, the cell encapsulated hydrogels were penetrated with 0.1 per cent Triton X-100 (Sigma-Aldrich, Germany) in PBS for 15 min for intracellular staining. After rinsing with PBS/0.1% Triton, the cells were washed and blocked with 10% goat serum for 30 min at room temperature. Three times washing with PBS were done, and essential antibodies against cardiac troponin T (TNT) (2.5 µg/mL; Abcam plc, Cambridge, UK) and GATA4 (2 µg/mL; Biorbyt, Cambridge, UK) was used for 24 h at 4 °C with cell encapsulated hydrogels. The cell encapsulated hydrogels were incubated for 1 h at 37 °C in a dark place with secondary antibodies goat anti-mouse IgG, Alexa Fluor^®^ 488 conjugate to TNT detection (5 g/mL; Biorbyt, UK) and goat anti-rabbit IgG, Fluorescein isothiocyanate (FITC) intermix to GATA4 detection (22 µg/mL; Sigma-Aldrich GmbH, Hamburg, Germany). PBS/1% BSA was used to dilute the antibodies. Cell nuclei were marked in PBS with 1 g/mL diamidinophenylindole (DAPI; Roche GmbH, Mannheim, Germany) for 20 min at 25 °C in a dark place after washing with PBS. Hydrogel-cell mixes were coated with mounting media for fluorescence microscopy (Ibidi GmbH, Munich, Germany).

Fluorescence signals were identified using computer-guided fluorescence microscopy (Axio Observer and AxioVision Rel.4.8; Zeiss MicroImaging GmbH, Jena, Germany) [53]. For Alexa Fluor^®^ 488 conjugated antibodies, the excitation and emanation wavelengths were 495 nm and 519 nm, individually.

### 4.6. Statistical Analysis

The information in this research was studied via one-way ANOVA analyses. The Tukey–Kramer post-hoc GraphPad Prism Software (V.6) was utilised to define a statistical significance among samples. The *p*-value < 0.05 was determined as statistically significant.

## Data Availability

The data presented in this study are available on request from the corresponding author. The data are not publicly available because it also forms part of an ongoing study.
